# Blood Vessel Resident Human Stem Cells in Health and Disease

**DOI:** 10.1093/stcltm/szab001

**Published:** 2022-02-19

**Authors:** David J Craig, Aaron W James, Yiyun Wang, Manuela Tavian, Mihaela Crisan, Bruno M Péault

**Affiliations:** 1 Center for Regenerative Medicine, University of Edinburgh, Edinburgh, UK; 2 Center for Cardiovascular Science, University of Edinburgh, Edinburgh, UK; 3 Department of Pathology, Johns Hopkins University, Baltimore, MD, USA; 4 INSERM, IRFAC/UMR-S1113, Strasbourg, France; 5 Orthopaedic Hospital Research Center and Broad Stem Cell Research Center, University of California, Los Angeles, Los Angeles, CA, USA

**Keywords:** hematopoiesis, mesenchymal stem cell, pericyte, tunica adventitia, fibrosis, vascular endothelium

## Abstract

The vascular wall is comprised of distinct layers controlling angiogenesis, blood flow, vessel anchorage within organs, and cell and molecule transit between blood and tissues. Moreover, some blood vessels are home to essential stem-like cells, a classic example being the existence in the embryo of hemogenic endothelial cells at the origin of definitive hematopoiesis. In recent years, microvascular pericytes and adventitial perivascular cells were observed to include multi-lineage progenitor cells involved not only in organ turnover and regeneration but also in pathologic remodeling, including fibrosis and atherosclerosis. These perivascular mesodermal elements were identified as native forerunners of mesenchymal stem cells. We have presented in this brief review our current knowledge on vessel wall-associated tissue remodeling cells with respect to discriminating phenotypes, functional diversity in health and disease, and potential therapeutic interest.

## Graphical Abstract

**Graphical Abstract:**
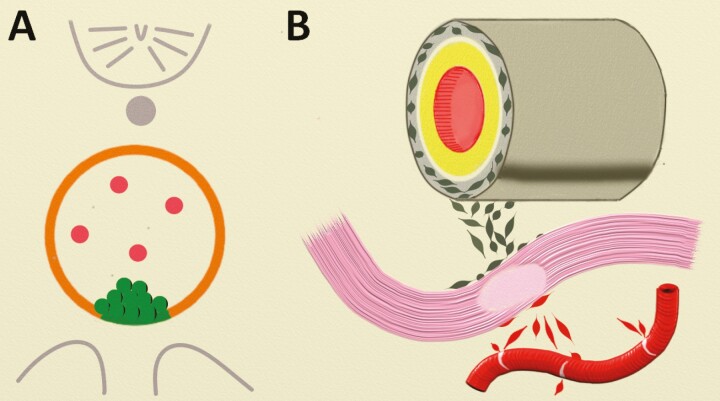
Contribution of blood vessel walls to non-vascular tissue development and regeneration. (A) In the embryo, definitive, adult-type hematopoietic stem cells and progenitors (green spheres) are produced by a subset of blood-forming endothelial cells that operate transiently in the ventral aspect of the aorta (orange) and vitelline arteries. (B) In the developed organism, pericytes (red) detached from microvessels, and adventitial cells (dark grey) migrating from larger arteries and veins can be mobilized to sites of tissue injury and contribute to scarring (fibrosis) and regeneration.

**Significance Statement**

In this article, we give a concise overview into the history of blood vessel resident human stem cells and their roles in health and disease. We first describe basic blood vessel anatomy and function and highlight the cells of the vascular stem cell niche and their respective contributions to endogenous tissue regeneration, use in cell therapies, and in tissue engineering.

## Introduction

From the ancient Greeks to the modern day, philosophers, physicians, and scientists have endeavored to understand the role of blood vessels and uncover their hidden functions. The Greek physician Praxagoras believed that blood vessels were channels that carried *pneuma*, the “life source” throughout the body,^1^ whereas the Byzantine author Meletius the Monk proposed in the 8th century that liver veins turn body humors into blood, hence are directly responsible for the “construction of man” (*de hominis fabrica*).^2^ Since then we have come to understand that blood vessels are conduits through which blood containing nutrients and oxygen is circulated to maintain tissue and organ health and homeostasis. William Harvey, a 15th-century physician, is usually credited as the first to give a detailed description of the systemic circulation and the function of the heart to pump blood throughout the body to different organs and tissues.^3^ However, in more recent years and perhaps in a twist of fate, we have come to find that blood vessels do indeed contain a “life source” of sorts in the form of resident stem cells which participate in tissue development and turnover, and have emerged as key candidates for regenerative medical therapies and in the developing field of tissue engineering. Such blood vessel resident multi-lineage mesodermal progenitor cells may, however, also mediate severe pathogenic processes such as vascular calcification, atheroma plaque formation, and fibrosis. Beyond a refined inventory of vascular cell types, this brief review stresses the importance of documenting the phenotypic and functional diversity of progenitor cells present in vascular walls.

## Blood Vessel Anatomy: Distinct Vascular Niches and Functions

There are 5 major classifications of blood vessels in the systemic circulation. Oxygenated blood is pumped from the heart to arteries that branch and narrow to arterioles. Arterioles in turn lead to capillary beds which are the major site of oxygen and nutrient exchange in organs and tissues. Deoxygenated blood is then carried from venules to veins back to the heart. The size, anatomy, and cellular constituents of the blood vessel niche may differ depending upon their primary function and the blood pressure they are exposed to.

The 3 layers of the vessel wall common to arteries and veins are the *tunica intima*, *tunica media*, and *tunica adventitia*. The tunica intima is the innermost layer of blood vessels and is comprised of a continuum of single-layered endothelium stretching throughout all blood vessels and the inner layer of the heart. In the intimal layer of arteries, the endothelium is supported by the internal elastic lamina separating the intima from the tunica media. The tunica media is comprised of smooth muscle and elastic fibers. Separating the medial and adventitial layers is the external elastic lamina. The adventitial layer of blood vessels consists of collagen fibers and fibroblast-shaped cells and primarily provides support to the vessel. The size of each layer differs between vessel types, with arteries primarily having larger medial and adventitial layers than veins.

Cells in vascular niches have specific roles in the regulation of blood vessel function. From the initial description in the 1860s until the 1970s, endothelial cells were primarily thought of as a mere physical barrier that separates blood and circulating cells and molecules from the surrounding tissue.^4^ In fact, due to this location within the vessel wall the endothelium also acts to control vascular tone through the regulation of vasoconstriction and vasorelaxation.^5^ However, vascular tone can be controlled in either endothelial-dependent or independent manner.^6^ Subsequently by regulating vascular tone endothelial cells directly influence blood flow and the leakage of hormones, solutes, and blood cells into surrounding tissues. A host of cell surface and intracellular markers can be used to distinguish endothelial cells from other cells within the perivascular niche. Some examples of human endothelial cell markers include CD31 (PECAM-1), CD34, CD144 (VE-cadherin), CD146 (MCAM, S-endo1), and von Willebrand factor (vWF).^7^ Some of these markers, such as CD34 and CD146, are not specific and shared with other perivascular and non-vascular cell types.

In arteries, vascular smooth muscle cells (vSMCs) constitute the majority of the tunica media and in healthy individuals have a contractile phenotype which allows them to regulate blood vessel diameter and by extension blood pressure. In the healthy vessel wall, vSMCs play a key role in extracellular matrix production through the secretion of elastin, collagen, and other proteoglycans.^8^ vSMCs are typically identified via the expression of the contractile proteins α-smooth muscle actin (αSMA) and smooth muscle myosin heavy chain (myosin 11, MYH11) and by their long spindle-like morphology.^9,10^ However, in response to changes in their environment, vSMCs may undergo alterations to morphology and the expression of cellular markers.^11^ Changes in vSMC from a contractile to a synthetic phenotype is an essential pathological mechanism underlying atherosclerosis.^12^

Adventitial cells (aka adventicytes), as the name suggests, reside in the tunica adventitia of the vessel wall. Initially, the adventitial layer has been regarded as a combination of fibroblasts, collagen, and nerves maintaining vessel structural integrity. However, in recent years, the role of the adventitial layer has been revealed as more intricate and complicated, with cells of the adventitia found to be key players in immunomodulation, the inflammatory response, and vessel remodeling.^13,14^ Adventicytes in vivo express CD34 but lack expression of the endothelial cell markers CD31 and CD146 as well as the hematopoietic cell marker CD45.^15,16^ Cells of the adventitial layer have also been described as providing a niche signaling environment to support stem and progenitor cells. In adult mice, Sca1^+^ progenitor cells were found to exclusively inhabit the adventitia in the vessel wall and give rise to SMCs in vitro.^17^ A second population of Sca1^+^ adventitial cells may also differentiate into macrophage-like cells.^18^ In the aortic root and thoracic aorta sonic hedgehog (SHH) signaling regulates adventitial progenitor cell proliferation, self-renewal, and survival.^19^ During late embryonic development and early neonatal growth Sca1^+^ adventitial progenitor cells upregulate SHH signaling reporters such as Gli1.^19^

The term “mural cells” refers to the pericytes of the microvasculature. These are named as such due to their position within the perivascular niche, lining endothelial walls. Pericytes, first described by Rouget in the 1870s, are found enveloping endothelial cells in capillaries.^20^ The defining characteristics of what constitutes a pericyte vary somewhat between authors. However, in vivo a pericyte can be described as having an elongated morphology, enveloping endothelial cells in the microvessel wall, and having protrusions embedded in the basement membrane shared with endothelial cells.^21^ Although believed to only be found in capillaries and small vessels, populations of pericytes were observed in the sub-endothelial layer of arteries and the *vasa vasorum*.^22,23^ In terms of function, pericytes regulate microvascular blood flow via alterations in capillary relaxation and contraction similarly to their large vessel counterparts, vSMCs. Pericytes are heterogeneous cells with numerous subpopulations being currently identified, as described below. Common pericyte markers include PDGFRβ, CD146, αSMA, neuron-glial antigen 2 (NG2), desmin, and CD13. Expression of these markers may depend upon the tissue in which the pericytes reside and their function within that tissue.

Aside from their roles in maintaining vessel homeostasis and function, cells of the blood vessel wall regulate vascular development. The growth of new blood vessels from the existing vasculature, in the process of angiogenesis, is mediated primarily following the binding of platelet-derived growth factor B (PDGFB) to PDGF receptor β (PDGFRβ). Endothelial cells release PDGFB which then attracts pericyte progenitor cells expressing PDGFRβ to the newly forming vessel, conferring stability, and promoting vessel integrity.^24-26^ Much of the information regarding this process has been gathered from the study of genetically modified mice. Germline ablation of PDGFB or PDGFRβ results in embryo death due to severe hemorrhaging meaning that this interaction between endothelial cells and pericytes is essential for the development of an organism.^26^ The association between pericytes and endothelial cells further requires the retention of PDGFB in the perivascular space.^27^ Cells of the vessel wall work in concert during vascular development and the maintenance of vascular homeostasis. However, in recent times, a bulk of research has focused on the vessel wall as a source of stem cells with possible applications in regenerative medicine.

## Blood Forming Endothelial Cells

**Figure 1.**
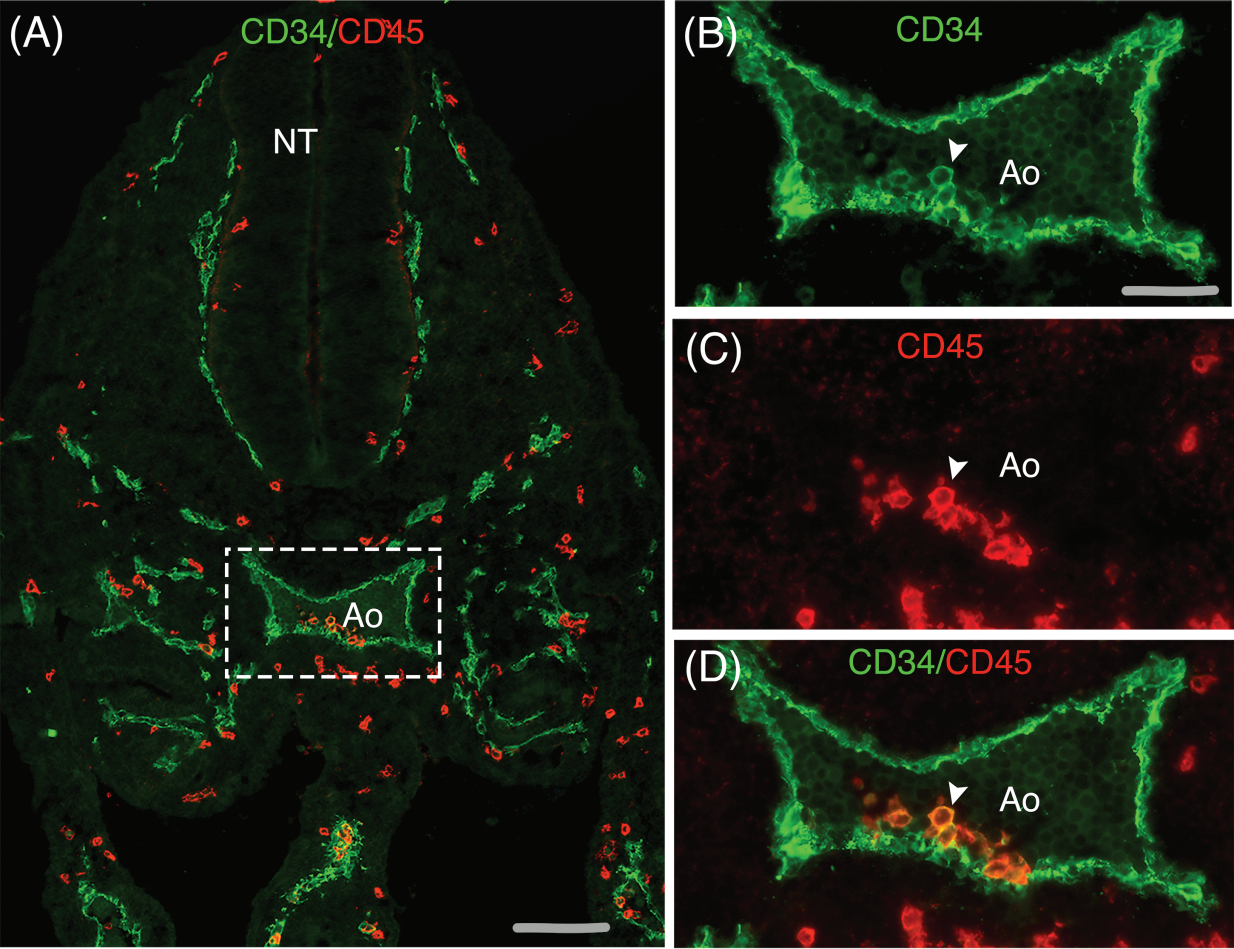
Hemogenic endothelium in the human embryo. (A) Transverse section through a 32-day human embryo stained with anti-CD34 (green) and anti-CD45 (red) fluorescent antibodies. The vascular system is conspicuously outlined by endothelial CD34 expression, and hematopoietic cells of extraembryonic origin are diffusely distributed within embryonic tissues (×10). (B-D) Higher magnifications of the dorsal aorta (Ao) region framed in A. Arrowhead points to a cluster of hematopoietic stem cells sprouting from the ventral endothelium of the aorta, some of which still co-express CD34. Scale bar = 80 µm. NT, neural tube.

Embryonic development requires permanent tissue oxygenation by erythroid cells, and a vascular network to dispatch these red blood cells. Hence, endothelial and blood cells are the first terminally differentiated elements that emerge in early ontogeny, and hematopoiesis adapts to the rapidly changing anatomy of the mammalian embryo and fetus by proceeding successively in the yolk sac and liver before being stabilized in bone marrow till adult life.^28^ The notion that the extraembryonic yolk sac, as the earliest provider of blood cells, is also the source of stem cells for lifelong hematopoiesis was first challenged in experimental avian chimeras.^29^ Instead, the region of the mammalian embryo encompassing the aorta, gonads, and mesonephros (AGM) was recognized at the very origin of definitive hematopoietic stem cells (HSCs).^28,30,31^ As early as 1917, F. Sabin had proposed that both endothelial and hematopoietic cells emerge in the chicken yolk sac from a common progenitor cell,^32^ although the existence of such “hemangioblasts,” as these were later named, was only documented functionally 80 years later.^33^ Sabin suggested that blood cells also sprout from preexisting endothelial cells in yolk sac vessels.^32^ This concept of an “hemogenic endothelium” was later fully validated in the AGM, where ventral aortic endothelial cells transiently produce HSCs.^30,34,35^ In human gestation, clusters of HSCs and other hematopoietic progenitors are seen adherent to endothelial cells on the ventral aspect of the dorsal aorta and vitelline artery between 27 and 40 days of development^28,36,37^ (Fig. 1). The blood-forming potential of endothelial cells from the human AGM was confirmed in culture.^38^ It was also demonstrated in the human embryo that only HSCs derived from the AGM—and not the extraembryonic yolk sac—have full lympho-myeloid definitive hematopoietic potential.^39^ In sum, many decades of research have led to the conclusion that some embryonic vessel walls include a minor subset of endothelial cells of splanchnopleural origin that are committed to hematopoiesis, and represent the ultimate source of the hundreds of billions of blood cells produced daily in the human body. Interestingly, recent data indicate that the avian and murine embryonic/fetal bone marrow also contains hemogenic endothelial cells that function in the perinatal period, albeit at a low frequency.^40^ Whether blood-forming intimal cells persist throughout life to be mobilized in case of severe hematopoiesis deficiency, for instance after irradiation, remains to be explored.

## Mesenchymal Stem Cells From Blood Vessels

Originally, the acronym MSC stands for “mesenchymal stem cell,” referring to a population of bone marrow-derived cells that can be isolated by adherence to plastic in culture and differentiate into chondrocytes, adipocytes, and osteoblasts,^41^ all characteristics of cells derived from embryonic mesodermal tissues.^42^ Since then the terms “mesenchymal progenitor cells,” “multipotent adult progenitor cells,” “mesenchymal stromal cells,” and “multipotent stromal cells” have all been used to describe the same population of cells and also introduce the idea that these cells may originate in the stromal cell compartment in situ.^43,44^ As well as the aforementioned ability to adhere to plastic, undergo tri-lineage differentiation, and the capacity for sustained proliferation, further criteria to identify MSCs in vitro include the expression of surface antigens: CD105, CD90, and CD73 and the absence of CD45, CD19, CD14 CD11b, CD34, CD79α, and HLA-DR.^45^

**Figure 2.**
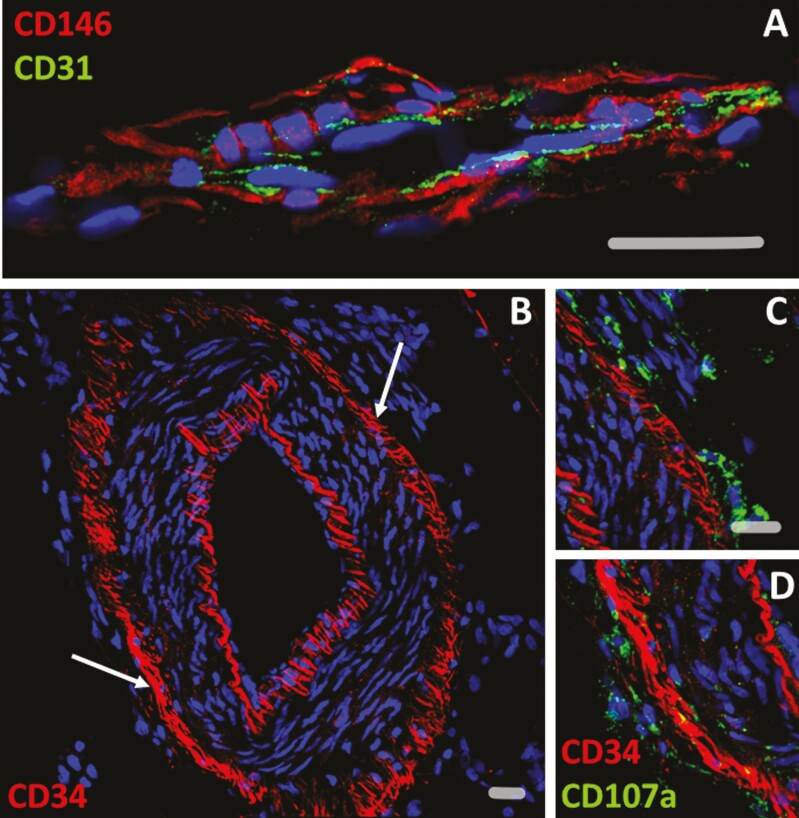
Presumptive mesenchymal stem/progenitor cells in human adipose tissue blood vessel walls. (A) Pericytes marked by CD146 expression (red) ensheath a microvessel seen in sagittal section. Endothelial cells are typified by CD31 expression (green). (B) Transverse section through an artery. Endothelial cells expressing CD34 (red) line the vessel lumen. In addition, non-endothelial CD34^+^ cells constitute a ring-like structure in the vascular tunica adventitia (arrows). (C, D) higher magnifications (arrows) of the tunica adventitia seen in B, co-stained with an antibody to CD107a. CD107a^+^ cells are seen outside and concentric to CD34^+^ adventitial cells (C), whereas some co-express CD34 in the adventitia (D). Scale bar = 20 µm.

For many years after the term MSC was coined, the in situ counterpart of these cells remained elusive. After bone marrow, adult adipose tissue was next identified as a major source of MSCs and subsequent studies found that MSCs can be isolated from virtually all vascularized organs in humans and mice. However, the question remained, which cells within organs give rise to MSCs in vitro? Phenotypic similarities between pericytes and MSCs have been observed.^46,47^ We provided the first evidence that human pericytes isolated by flow cytometry from fetal and adult tissues, including the heart and skeletal muscle become a homogenous population of MSCs in culture,^48^ as assessed by morphology, phenotype, rate of proliferation, and developmental potential. In that study, pericytes were identified on the expression of CD146, NG2, PDGFRβ, and the absence of endothelial, myogenic, and hematopoietic cell markers (Fig. 2). These cells also adhere to plastic, undergo tri-lineage differentiation, and display the canonical MSC markers CD73, CD90, and CD105.^48^ Thus, an in situ identity of the MSC had been revealed. Subsequently, we identified adventitial cells from human adipose tissue as a second parent cell for MSCs: CD34^+^ adventitial cells lacking the expression of CD31, CD146, and CD45 (Fig. 2) display MSC markers in situ and once cultured give rise to a clonogenic population of MSCs that can differentiate into osteocytes, chondrocytes, and adipocytes.^15^ Collectively pericytes and adventitial cells have come to be known as perivascular stem cells, or PSCs, even though these 2 classes of cells appear to play complementary, yet distinct roles in tissue repair.^49^

It is essential to note that perivascular cells are not reprogrammed into repair cells exclusively in culture, but may be naturally involved in tissue turnover and regeneration. Indeed, the transition from perivascular cells to MSCs in vitro reflects an intrinsic tissue regenerative potential of these cells, even though the existence in vivo of a cell functionally identical to the cultured MSC has not yet been demonstrated. Dellavalle et al documented a role for pericytes in skeletal muscle regeneration and satellite cell replenishment following cardiotoxin acute injury.^50^ Resident mural cells are progenitors of white adipocytes^51^ and follicular dendritic cells^52^ in situ. Adventitial cells and pericytes have been described at the origin of fibrosis in several pathologies,^53-57^ and are essential players in dental pulp turnover and regeneration.^58^

## Perivascular Stem Cell Heterogeneity

Despite expressing ubiquitous markers and playing common roles in all organs, perivascular cells are heterogeneous populations, the diversity of which only begins to be uncovered. In the kidney, for instance, mesangial cells in glomeruli are modified pericytes,^59^ as are renin-producing cells in afferent juxtaglomerular arterioles.^60,61^ Regarding tissue repair, we and others have demonstrated that perivascular cells—similar to their MSC progeny—play multiple roles: progenitor activity, inflammation modulation, stem/progenitor cell support, angiogenesis stimulation, and scarring. This suggests the existence of discrete, functionally distinct PSCs. Indeed, we found in skeletal muscle that PDGFRβ^+^ PDGFRα^−^ cells are myogenic, while PDGFRβ^+^ PDGFRα^+^ cells are fibro-adipogenic progenitors.^62^ As to cell lineage differentiation, pericytes/MSCs expressing the ROR2 Wnt receptor have a higher chondrogenic potential than ROR2-negative counterparts.^63^ A subset of EphA7 expressing, capillary-associated mouse pericytes have been recently described as multipotent progenitors endowed with high angiogenic potential.^64,65^ We also found that mural osteogenic cells can be mapped by PDGFRα and CXCR4 expression.^66,67^ We observed, in addition, that whole pericytes or adventicytes can be segregated into ALDH (aldehyde dehydrogenase)^bright^ and ALDH^dim^ subsets, the former including more primitive progenitors.^68^ We have used cell surface antigen screens as well as transcriptomes and tissue microarrays to identify novel markers of human pericyte and adventicyte subsets.^69^ We observed that CD10, a marker of normal and leukemic hematopoietic cells, typifies a subpopulation of perivascular osteoprogenitors devoid of adipogenic and other potentials.^70^ Conversely, CD107a (aka LAMP1) is expressed by human adventicytes with high propensity to differentiate into adipocytes, whereas CD107a^−^ cells are enriched in osteogenic progenitors^71^ (Fig. 2). In summary, it is clear that even within “pure” mesenchymal constituents of vessel walls, such as pericytes and adventicytes, exists an important cellular heterogeneity. Distinct degrees of “stemness” have been identified among perivascular cells in adipose tissue,^68^ and diversely lineage-committed progenitors coexist within these niches, suggesting the existence of a hierarchy of perivascular mesenchymal progenitor cells. It remains to be determined whether this ranking exists in all organs, and to what extent these progenitors are involved in tissue development, turnover, and remodeling.

Our understanding of the mode of action of MSCs in tissue repair has evolved from cell-for-cell replacement by these multipotent progenitors to the secretion of diverse regeneration supporting growth factors and cytokines.^72^ Assessing the production of trophic factors by functionally defined subsets of PSCs is a priority, encouraged by the demonstration that cells from the intact stromal vascular niche secrete a much larger spectrum of such factors than their cultured MSC descent.^73^

## Perivascular Cells in Pathology

### Vascular Remodeling/Atherosclerosis

Atherosclerosis is a cardiovascular disease were the buildup and hardening of plaque in arterial walls leads to narrowing of the artery, limiting blood flow. Risk factors for atherosclerosis include aging, hypertension, hypercholesterolemia, and obesity.^8^ Underlying pathogenic processes in atherosclerosis involve lipoprotein accumulation in the arterial wall, endothelial activation, and dysfunction as well as activation of the immune system. Monocytes and macrophages infiltrated into the vessel wall consume plaque cells and become *foam cells* which make up the atherosclerotic plaque. Cell fate genetic mapping has demonstrated the contribution of resident medial vSMCs to neointima formation and thickening.^74^ vSMCs may also switch to a de-differentiated “synthetic” phenotype and form the fibrous cap of the atherosclerotic plaque to stabilize it.

Perivascular MSC progenitors have been implicated at different stages of atherosclerosis. Pericyte-like cells accumulate in the aortic intima during atherosclerosis,^75^ and pericytes residing in the sub-endothelial layer of arteries contribute to atherosclerotic plaque formation.^22,23^ Cells of the adventitial layer may also play roles in the formation of atherosclerotic lesions. The *tunica adventitia* contains non-SMC progenitor cells also involved in neointima formation and typified in mice by Sca-1 expression.^17,19,76^ Mouse adventitial cells expressing Gli1 develop into MSCs in culture and contribute to atherosclerosis in vivo, by differentiating into both SMCs and osteoblast-like cells.^77,78^ Moreover, correlations between adventitial inflammation and intimal lesion formation have been observed for many years. In *ApoE*^*−/−*^ mice, a commonly used model of atherosclerosis, adventitial tertiary lymphoid tissues were observed.^79^ Although contribution to atherosclerotic plaques is not fully understood, these ectopic structures have been assumed to mediate antigen clearance and immune activation.^80^ The prominent and ubiquitous osteogenic potential of human PSCs^81^ suggests a role for pericytes and adventitial cells in ectopic calcification, which may also occur in atheromatous blood vessels. Finally, angiogenesis within the vessel wall also plays a role in atherosclerotic disease progression, and pericytes were found to be recruited to forming vessels of the atherosclerotic plaque via the release of hepatocyte growth factor.^82^

### Tissue Fibrosis

Transient fibrogenesis is crucial for tissue healing after acute injury, while chronic fibrogenic response results in irreversible degeneration due to the replacement of damaged tissue with myofibroblasts producing excessive amounts of extracellular matrix. Several studies relying on cell lineage tracing in mice have given a central role to pericytes at the origin of myofibroblasts. Kidney ischemia-reperfusion drives the generation of myofibroblasts by FoxD1^+^ stromal cells expressing the pericyte markers PDGFRβ and CD73,^54^ while acute skin and muscle injuries activate ADAM12^+^ pericytes that differentiate into myofibroblasts.^55^ Similarly, pericytes co-expressing PDGFRα and PDGFRβ are at the origin of the fibrotic scar developing after spinal cord injury,^83^ and myofibroblasts populating the acutely injured liver derive from stellate cells, which are specialized pericytes.^84^ We have recently tracked the transition from perivascular cells to myofibroblasts in post-traumatic osteoarthritis, using either PDGFRα or PDGFRβ as a marker.^85,86^ Perivascular pro-fibrotic cells were also identified in diverse organs by expression of the Gli1 transcription factor,^56^ and pharmacologic inhibition of Gli1^+^ perivascular cells in the bone marrow reduces fibrosis.^87^ Moreover, we have used PDGFRβ-Cre × mTmG reporter mice to track pericytes following acute skeletal and cardiac muscle injury. PDGFRβ-Cre effectively targeted recombination in quiescent PDGFRβ^+^ pericytes, as well as activated myofibroblasts populating fibrotic lesions.^88^ This collection of data using diverse perivascular reporter systems demonstrates that select perivascular cell types across organ systems have clearly defined roles in tissue fibrosis.

Besides fibrosis and pathologic vascular remodeling, pericytes have been involved in melanoma cell migration along the abluminal aspect of blood vessels (pericyte mimicry),^89,90^ PDGFRβ signaling promoting breast cancer metastasis to the brain,^91^ and in the development of perivascular soft tissue tumors.^92^

## Conclusion: Blood Vessel-Borne Stem Cells for Cell Therapy and Tissue Engineering

The best-characterized developmental affiliation between vascular cells and a different cell lineage links hemogenic endothelial cells and definitive HSCs, within an anatomically and timely very restricted window of mammalian ontogeny. While this biological mechanism has offered a unique model to decipher the molecular control of hematopoiesis incipience,^93^ such an ephemeral population of a few hundred cells present in the first-trimester human embryo appears to be devoid of any therapeutic significance. However, human hemangioblasts and hemogenic endothelial cells have been successfully and efficiently derived from human pluripotent stem cells,^94-96^ opening the possible use of these angio-hematopoietic cells in a restorative setting. Remotely from hematopoiesis, we have also described in skeletal muscle a population of myogenic endothelial cells,^97^ suggesting the contribution of the tunica intima to other cell lineages.

More widely distributed—in fact, ubiquitous in pre- and postnatal organs—are PSCs, including both pericytes and adventicytes. Experimentally, purified PSCs have been used to engineer blood vessels,^98^ heal skin wounds,^99^ and regenerate lung,^100^ skeletal muscle,^48,101^ cartilage,^15,48,102-104^ ischemic limbs,^105^ tendons,^106^ uterus,^107^ heart,^108,109^ and Leydig cells in the testis.^110^ In particular, PSC osteogenic potential has been documented in great detail, in culture, and in vivo (reviewed in 81). Selected, well-characterized perivascular cells offer multiple advantages over their long-term cultured MSC progeny. Important differences exist in the healing potential of MSCs produced by different laboratories, batch-to-batch variations being attributable to donor demographics and differences in cell processing and culture conditions. Moreover, conventional MSCs are derived from total, unselected cell suspensions, and hence from mixed cell populations undergoing in culture substantial changes in gene expression, as well as dramatic clonal selection,^111^ which may result in unpredictable phenotypic and functional heterogeneity.^72^ Cell culture may also, albeit rarely, induce hazardous genetic abnormalities: in animals, transplanted MSCs formed occasionally malignant tumors in the infarcted heart^112^ and arthritic joint,^113^ adding to MSC intrinsic propensity to provide stromal support for tumor growth.^114^ The use of prospectively identified perivascular pre-MSCs, in the absence of expansion in culture, may alleviate such issues. On the other hand, characterization of an array of diversely committed PSCs may also considerably improve MSC production. The balance, during MSC-driven tissue regeneration, between progenitor activity, growth factor and cytokine secretion, support of local stem cells, and immunomodulation has not been properly evaluated.^115^ Such diverse functions suggest the existence of distinct, specialized MSC subsets that could be selected a priori for superior therapeutic efficacy. The ongoing identification of functionally committed subsets of perivascular MSC ancestors may serve this goal. Along this line, MSCs could be properly “tuned” for the intended therapy, representing a form of “personalized medicine” while currently a single method is used to produce MSCs to treat conditions as diverse as GvH disease, myocardial infarcts, or musculoskeletal injuries.^72^

## Data Availability

No new data were generated or analyzed in support of this research.
